# A Randomized Phase II/III Trial Evaluating the Efficacy and Safety of 100 and 125 µg of Calcifediol Weekly Treatment of Severe Vitamin D Deficiency

**DOI:** 10.3390/nu17040672

**Published:** 2025-02-13

**Authors:** Jose Luis Pérez-Castrillón, Esteban Jódar-Gimeno, Ján Nociar, Michal Lojka, Dimitar Nikolov, Fernando Cereto-Castro, Snežana Novković, Umberto Tarantino, Nadia Mehsen-Cetre, Paula Arranz, Cristina Martínez Ostalé, Aintzane García-Bea, Inmaculada Gilaberte

**Affiliations:** 1 Department of Internal Medicine, Río Hortega University Hospital, 47012 Valladolid, Spain; 2 Department of Endocrinology and Nutrition, Quirón Madrid University Hospital, Universidad Europea, 28670 Madrid, Spain; 3 Department of Cardiology, General Hospital with Polyclinic Lučenec n.o., 98401 Lučenec, Slovakia; 4 Ordinace MediFem, s.r.o., 41501 Teplice, Czech Republic; 5 Department of Rheumatology, Medical Center-1-Sevlievo, 5400 Sevlievo, Bulgaria; 6 Department of Internal Medicine, Hospital Quirón Barcelona, 08023 Barcelona, Spain; 7 Department of Internal Medicine, Institute of Rheumatology, 11000 Belgrade, Serbia; 8 Department of Orthopaedics and Traumatology, Policlinico Tor Vergata Foundation, 00133 Rome, Italy; 9 Service de Rhumatologie CHU Pellegrin-Tripode, 33000 Bordeaux, France; 10 Clinical Research Department, FAES FARMA, 48940 Leioa, Spain; 11 Medical Affairs Department, FAES FARMA, 48940 Leioa, Spain

**Keywords:** calcifediol, vitamin D deficiency, efficacy, safety, weekly treatment

## Abstract

**Background/Objectives:** Given the crucial health benefits of vitamin D, addressing severe deficiencies is a pressing medical concern. This study aimed to evaluate the effectiveness and safety of two new weekly doses of calcifediol (100 µg and 125 µg) for long-term management in patients with severe vitamin D deficiency, defined as plasma 25(OH)D levels ≤10 ng/mL. **Methods**: This study was a randomized, two-cohort, controlled, double-blind, multicentre phase II–III trial. Subjects were randomized 2:2:1 to weekly calcifediol 100 µg, 125 µg or a placebo. The primary endpoint was the proportion of patients achieving plasma 25(OH)D levels of ≥20 ng/mL and/or ≥30 ng/mL by week 16. **Results:** A total of 276 patients (mean age: 55.2 years, SD 15.42) were randomized. By week 16, 92.3% and 91.8% of patients in the calcifediol 100 µg and 125 µg groups, respectively, reached ≥20 ng/mL, compared to 7.3% in the placebo group. Levels of ≥30 ng/mL were achieved by 49% (100 µg) and 76.4% (125 µg) of participants, with none in the placebo group. Calcifediol demonstrated superior efficacy at all response levels and time points (*p* < 0.0001). Plasma 25(OH)D concentrations increased by week 24 and remained stable. The incidence of adverse events was comparable across groups. **Conclusions:** A weekly calcifediol dose of 100 µg demonstrates the best profile of efficacy and tolerability, providing a reliable solution for achieving and maintaining adequate vitamin D levels in patients with severe deficiency.

## 1. Introduction

Severe vitamin D deficiency is a common global issue. A recent global survey revealed that Latin America, Oceania, and North America have a prevalence ranging from 5% to 18% of this severe reduction in serum vitamin levels [1]. Moreover, the prevalence in Europe, Asia, and Africa is significantly higher, ranging from 24% to 49% [1].

Some factors, such as reduced sun exposure, latitude, high skin pigmentation, and some genetic polymorphisms, increase the risk of suffering vitamin D deficiency [1,2]. Additionally, severe vitamin D deficiency can be favoured by certain medical conditions such as cystic fibrosis, Crohn’s disease, and celiac disease, which lead to malabsorption [3], or by diseases affecting the kidneys and liver that reduce the amount of specific enzymes required to convert vitamin D into its active form [4].

Although there is some controversy surrounding optimal serum 25(OH)D levels for good health maintenance and therapeutic purposes, it is generally accepted that plasma levels of 25(OH)D below 10 ng/mL indicate severe deficiency [5]. This severe deficiency has been linked to metabolic bone diseases, including rickets in children and osteomalacia or osteoporosis in adults. Moreover, association studies have suggested links between vitamin D deficiency and extra-skeletal manifestations such as increased risk of musculoskeletal disorders, autoimmune diseases, infections, cardiovascular disease, diabetes mellitus, neurocognitive dysfunction, and several cancers, including colorectal, prostate or breast [6,7,8,9,10]. Based on this evidence, rapid correction with appropriate doses of vitamin D supplements is recommended [11,12,13,14].

Calcifediol (25-hydroxyvitamin D3, calcidiol) has been identified as the most potent supplement for increasing 25(OH)D levels in the treatment of vitamin D deficiency [15,16]. This potency is particularly clinically relevant for patients with severe deficiency, as rapid and effective repletion of vitamin D levels can be critical in improving their health outcomes [17]. Introducing a new weekly administration formulation offers significant advantages, as it can be more convenient for many patients, providing personalized alternatives, potentially increasing adherence to the treatment and its efficacy [18,19]. However, there remains a gap in our understanding of the optimal dosing regimen for weekly formulations that balances efficacy with safety, particularly in different populations, making it crucial to study new dosing strategies to refine treatment protocols and minimize potential side effects. This phase II/III, randomised, double-blind, placebo-controlled clinical trial was conducted to evaluate the efficacy of different strengths of weekly dose formulations of calcifediol. Patients were divided into two distinct cohorts based on their baseline plasma levels of 25(OH)D. In a previous publication [20], the efficacy and safety of weekly 75 µg and 100 µg calcifediol doses were demonstrated in patients with moderate vitamin D deficiency [10 ng/mL < 25(OH)D < 20 ng/mL]. The present article focuses on the efficacy and safety of long-term weekly administration of two new formulations of 100 µg and 125 µg of calcifediol compared to a placebo in the treatment of severe vitamin D deficiency.

## 2. Materials and Methods

This clinical trial was designed as a randomized, double-blind, two-cohort, double-dummy, multicenter study in phase II/III. Participants were assigned to one of two cohorts based on their initial 25(OH)D levels at visit 1: Cohort 1 (25(OH)D >10 to <20 ng/mL) and Cohort 2 (25(OH)D ≤ 10 ng/mL). The study took place across seven European countries from 28 December 2020 to 25 April 2023. Specifically, the trial included 7 centers in Bulgaria, 11 centers in the Czech Republic, 8 centers in Spain, 4 centers in France, 6 centers in Italy, 6 centers in Serbia, and 13 centers in Slovakia. Independent Ethics Committees provided approval for each site prior to the initiation of the study. Conducted in accordance with the Declaration of Helsinki, written informed consent was obtained from all participants before they were enrolled. The trial was registered on Clinicaltrials.gov (NCT04735926) and with EudraCT number 2020-001099-14. This manuscript reports the findings from Cohort 2.

### 2.1. Study Procedures

The schedule of the clinical trial is shown in Supplementary Table S1. After screening (visit 1), subjects were randomized to calcifediol 100 µg, calcifediol 125 µg or placebo at a ratio of 2:2:1 on visit 2. The treatment period began on the first Sunday following randomization and lasted for 52 weeks, with a subsequent 30-day follow-up. The subjects had to attend seven on-site visits (visits 1 to 7) and one telephone visit (follow up at visit 8). Calcifediol and placebo capsules were identical in size, colour, taste and appearance, and had to be administered orally once per week on Sunday morning during the treatment period. Blood sample extraction should always take place at least 48 h after the last IP intake. At weeks 16, 24, or 32, participants with 25(OH)D levels of ≤10 ng/mL received, along with the study treatment, daily oral cholecalciferol as rescue medication (Supplementary Table S3). Treatment adherence was assessed at visits 4, 6, and 7 by accounting and documenting unused capsules (including empty and partially empty containers).

### 2.2. Study Population

Subjects included in Cohort 2 of the study were males or females ≥18 years of age with vitamin D severe deficiency (serum 25(OH)D levels ≤ 10 ng/mL), who signed informed consent (see Supplementary Table S2 for a full description of eligibility criteria). Inclusion of patients with severe vitamin D deficiency was prematurely stopped by the sponsor. The rationale for stopping recruitment was that the study was designed pre-pandemic coronavirus disease (COVID-19). This pandemic impacted the recruitment for this study.

### 2.3. Endpoints and Assessments

The primary endpoint of the trial was the efficacy of weekly administration of two doses of calcifediol soft gelatine capsules (SGCs) compared to placebo assessed in terms of percentage of subjects who achieved 25(OH)D levels ≥20 ng/mL and/or ≥30 ng/mL at week 16 of treatment (visit 4). Participants with missing values, such as those who did not attend visit 4 (Week 16), were classified as non-responders in the primary analysis and excluded from the secondary analyses. Secondary endpoints encompassed the proportion of subjects reaching this response levels at weeks 4, 16, 24, 32, and 52; the measured 25(OH)D levels at these same time points; and the occurrence of a sustained response, defined as maintaining 25(OH)D levels of ≥20 ng/mL without reverting to a non-response status at subsequent visits. The safety assessments included the recording of treatment emergent adverse events (TEAEs), laboratory examinations, vital signs, weight, height, BMI, abdominal circumference, and physical examinations, and were assessed in all randomised patients who took at least one dose of trial medication (Safety Set).

Blood samples were analysed in a central laboratory (Supplementary Table S3).

### 2.4. Other Assessments

To further explore the previously defined blunted effect of obesity on 25(OH)D levels after vitamin D supplementation [21], participants were classified according to their body mass index (BMI) into four subgroups of obese (BMI ≥30 kg/m^2^), overweight (25 ≤ BMI < 30 kg/m^2^), normal weight (18.5 ≤ BMI < 25 kg/m^2^), and underweight (BMI < 18.5 kg/m^2^). Calcifediol 100 µg, 125 µg, and placebo treatments were evaluated for the proportion of subjects who reached 25(OH)D levels of ≥20 ng/mL and/or ≥30 ng/mL by week 16 in each BMI subgroup.

### 2.5. Statistical Evaluations

Sample size was determined employing nQuery (Supplementary Table S3), based on a previous study with calcifediol. It was estimated that 70 subjects for the placebo group and 140 subjects for each of the two calcifediol dosage groups would be required, totaling 350 subjects. Accounting for a possible discontinuation rate of up to 20%, the cohort size for randomization was projected to be 438 subjects. Randomization was conducted using the validated software SAS^®^ version 9.4 for Windows (SAS® Institute Inc., Cary, NC, USA; Supplementary Table S3).

Descriptive statistics were utilized to summarize continuous data by treatment group. The primary endpoint, along with the percentage of responders and all key secondary efficacy endpoints, was evaluated using a large-sample normal approximation test for proportions. To adjust for multiple comparisons, a Bonferroni correction was applied, setting the two-sided significance level at α = 0.0125 (0.05/4). Patients who received at least one dose of the investigational drug and had at least one 25(OH)D post-baseline assessment were included in the statistical analyses (full analysis set, FAS). Statistical significance was determined with a *p*-value <0.05. All statistical analyses were performed using SAS^®^ software within a validated and secure environment.

## 3. Results

**Figure 1:**
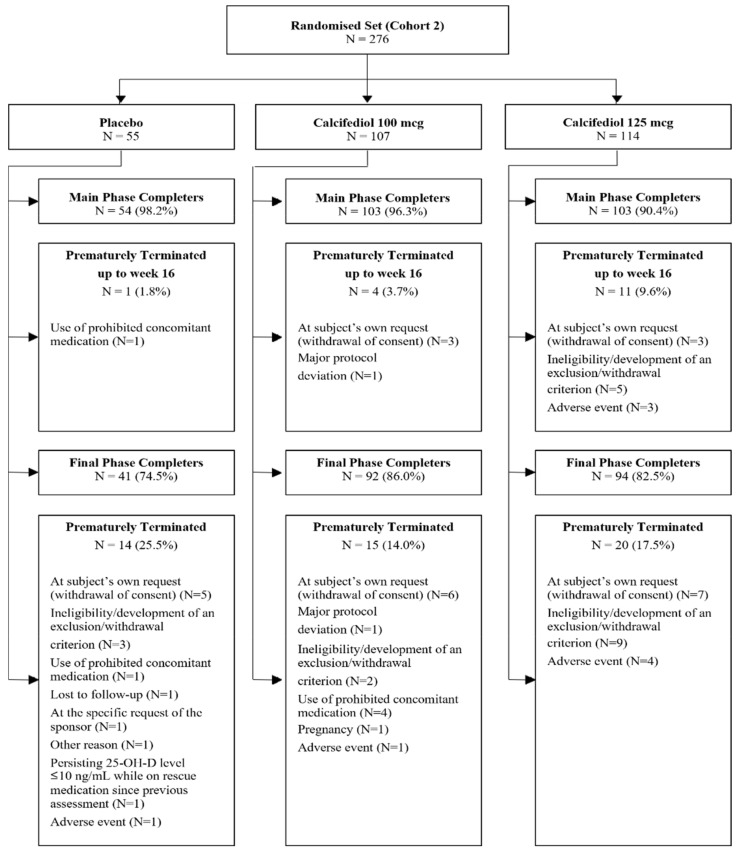
A patient disposition flow chart illustrating the number of randomized patients at each phase of the study. Reasons for early termination are also indicated.

A total of 276 subjects with baseline 25(OH)D levels ≤10 ng/mL were randomized (Figure 1). Of these, 260 participants successfully completed 16 weeks of treatment (main phase) and 227 subjects concluded the entire study (52 weeks).

The baseline demographic and clinical characteristics of the subjects were balanced among groups. Those of the population included in efficacy analysis (FAS population), which consisted of 55 subjects in the placebo group, 104 in the 100 µg of calcifediol group and 110 in the 125 µg calcifediol group, are shown in Table 1. The mean subjects’ age of the study population was 55.2 ± 15.4 years and 73.6% were female and 94.1% were white. The highest percentage of subjects by BMI classification was observed in the BMI subgroup ≥30 kg/m^2^ (112 subjects, 41.6%). The most common comorbidity in all groups was high blood pressure (49.4%), and the number of postmenopausal women ranged from 38.2% of patients in the placebo group to 47.1% in the calcifediol 100 µg group. There were not relevant differences between groups in baseline demographics and clinical conditions.

Most patients were included in the study during spring (52%), followed by winter (29%), when a higher incidence of vitamin D deficiency is expected due to a period of lower sunlight exposure in the Northern Hemisphere where this study was conducted. The percentage of subjects starting the study in different seasons was similar between treatment groups (Supplementary Table S4).

### 3.1. Efficacy of Calcifediol 100 µg and 125 µg After 16 Weeks of Treatment

**Figure 2:**
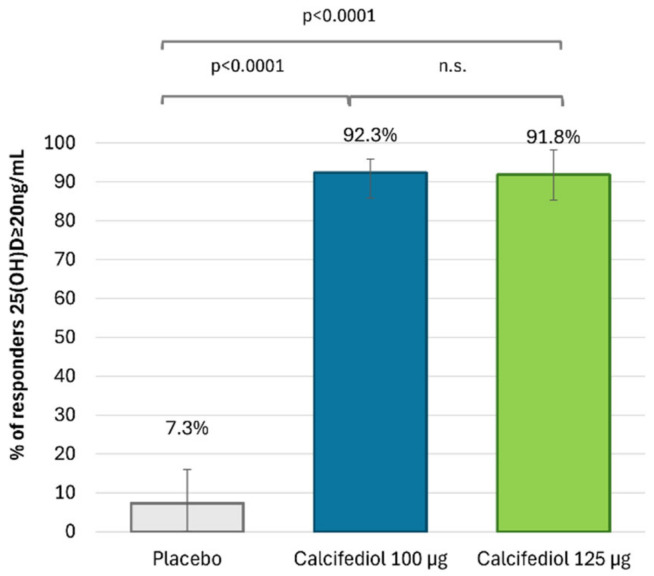
Proportion of individuals with 25(OH)D concentration ≥20 ng/mL at week 16 is shown for the placebo group (grey; n = 55), the 100 µg calcifediol group (blue; n = 104), and the 125 µg calcifediol group (green; n = 110). *p*-values from two-way analysis of proportions are provided. Error bars represent the 98.75% confidence intervals (CIs). n.s., non significant.

**Figure 3:**
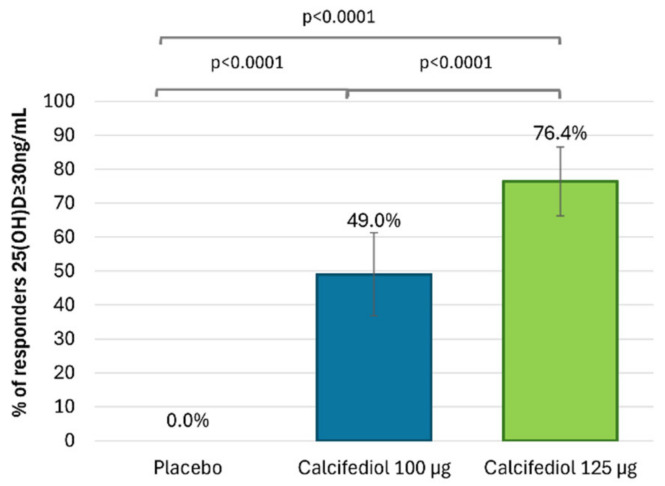
Proportion of individuals with 25(OH)D concentration ≥30 ng/mL at week 16 is shown for the placebo group (grey; n = 55), the 100 µg calcifediol group (blue; n = 104), and the 125 µg calcifediol group (green; n = 110). *p*-values from two-way analysis of proportions are provided. Error bars represent the 98.75% confidence intervals (CIs).

After 16 weeks of treatment, 96 (92.3%) individuals treated with calcifediol 100 µg, 101 (91.8%) with calcifediol 125 µg and 4 (7.3%) with a placebo presented 25(OH)D levels ≥20 ng/mL (Figure 2). The percentages of responders for a 25(OH)D response level of ≥30 ng/mL were 49.0% (51 patients) in the calcifediol 100 µg study arm, 76.4% (84 patients) in the calcifediol 125 µg and 0% in the placebo arm (Figure 3).

The superiority of both doses of calcifediol at each response level compared to the placebo was demonstrated (*p* < 0.0001). Both calcifediol 100 µg and 125 µg showed equivalent efficacy in the percentage of patients achieving a response concentration of ≥20 ng/mL. However, there were significant differences between calcifediol 100 and 125 µg to achieve a response concentration of ≥30 ng/mL at week 16.

Response rates of ≥20 and ≥30 ng/mL after 16 weeks of treatment were reevaluated within each treatment group after categorization by BMI. The BMI subgroup under-weight (BMI < 18.5 kg/m^2^) was omitted from the subgroup analyses, as only five subjects belonged to this category. The analysis revealed that the percentages of responders were not significantly different, regardless of BMI classification into normal-weighted (18.5 ≤ BMI < 25 kg/m^2^), overweighted (25 ≤ BMI < 30 kg/m^2^) or obese (BMI ≥ 30 kg/m^2^) subgroups (Supplementary Figure S1).

### 3.2. Efficacy of Calcifediol 100 µg and 125 µg over Time (Responders Rate)

**Figure 4:**
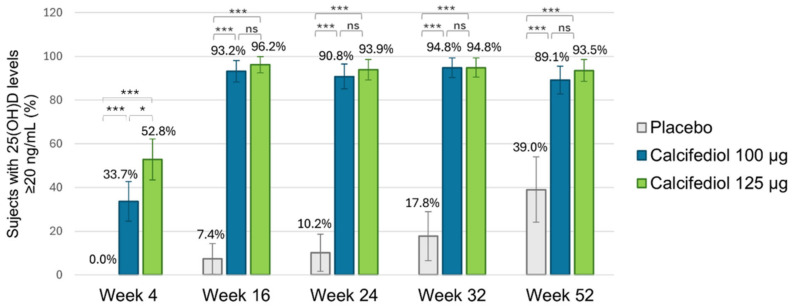
Proportion of individuals achieving a 25(OH)D concentration of ≥20 ng/mL is shown for the placebo group (grey; n = 55), the 100 µg calcifediol group (blue; n = 104), and the 125 µg calcifediol group (green; n = 110). *p*-values from two-sided comparisons of proportions are indicated (* *p* < 0.05, *** *p* < 0.0001, ns, non significant) and error bars represent the 98.75% CI (confidence intervals).

For a response level ≥20 ng/mL (Figure 4), response rates exceeding 90% were observed in both calcifediol groups from week 16 of treatment onwards (except for a response rate of 89.1% at week 52 in calcifediol 100 µg group), with no significant differences among calcifediol doses. Statistically significant response rate differences were observed for calcifediol 100 µg versus placebo and calcifediol 125 µg versus placebo at all visits (*p* < 0.0001).

It can be highlighted that out of the 16 subjects (39.0%) in the placebo group who reached a concentration ≥20 ng/mL at week 52, 13 subjects were receiving rescue medication.

**Figure 5:**
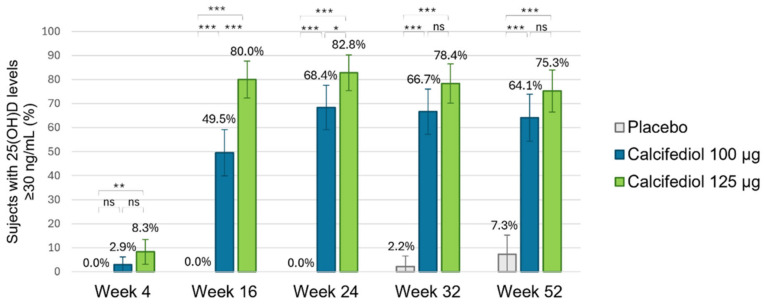
Proportion of individuals achieving a 25(OH)D concentration of ≥30 ng/mL is shown for the placebo group (grey; n = 55), the 100 µg calcifediol group (blue; n = 104), and the 125 µg calcifediol group (green; n = 110). *p*-values from two-sided comparisons of proportions are indicated (* *p* < 0.05, ** *p* < 0.001, *** *p* < 0.0001, ns, non significant) and error bars represent the 98.75% CI (confidence intervals).

For a 25(OH)D response level ≥30 ng/mL (Figure 5), response rates of >60% from week 24 onwards in the calcifediol 100 µg group and of >75% in the calcifediol 125 µg group from week 16 onwards were observed compared to a lower maximum response rate of 7.3% in the placebo group at week 52. Statistically significant response rate differences were observed for calcifediol 100 µg and 125 µg versus placebo at all visits. Statistical differences were found between calcifediol 100 µg and 125 µg doses in the percentage of responders ≥30 ng/mL at week 16, but this difference was reduced at week 24, and there were not significant differences thereafter in the proportion of responders ≥30 ng/mL in weekly calcifediol 100 µg and 125 µg groups.

### 3.3. The Proportion of Patients Who Achieved a Sustained Response

It is anticipated that 25-hydroxyvitamin D levels would fluctuate over the course of a year-long study, similar to variations in sunlight exposure, and that isolated increases in 25(OH)D measurements may not accurately indicate true recovery from deficiency. Therefore, it was assessed whether subjects who achieved response levels ≥20 ng/mL maintained this responder status over time (in subsequent visits until the end of the study) or if it was merely a transient rise in 25(OH)D levels. Most patients in the calcifediol groups of 100 µg (83 individuals, 79.8%) and 125 µg (93, 85.3%) had a sustained response throughout the study, with most responses from week 4 in the 125 µg calcifediol group and from week 16 in the 100 µg calcifediol group. A total of four subjects in the placebo group showed a maintained response ≥20 ng/mL starting at week 32, but all were receiving rescue medication. Thus, none of patients receiving only a placebo had a sustained response.

### 3.4. Subjects in Need of Rescue Medication

Patients with a 25(OH)D concentration ≤10 ng/mL after 16, 24 or 32 weeks of treatment were considered in need of rescue medication and were administered daily cholecalciferol 800 IU in addition to their trial medication until the end of the study. One subject of each calcifediol group, which corresponds to 1% of subjects in 100 µg calcifediol group and 0.6% in 125 µg calcifediol group, and a total of 30 subjects (54.5%) of the placebo group received rescue medication during the study. These subjects were considered in the efficacy analysis (full analysis set) to have a significant impact on the response results in the placebo group.

### 3.5. Evolution of 25(OH)D Concentration Throughout the Study

Mean (SD) 25(OH)D concentrations at baseline were similar among groups and equal to 8.07 (1.36) ng/mL in the placebo group, 7.93 (1.53) ng/mL in the 100 µg calcifediol arm, and 7.86 (1.66) ng/mL in the 125 µg calcifediol arm.

Starting from week 4, statistically significant differences in 25(OH)D levels were observed in both calcifediol groups compared to the placebo (*p* < 0.0001) at all assessments, with slightly significant differences (*p* < 0.02) between the levels of the two calcifediol groups.

**Figure 6:**
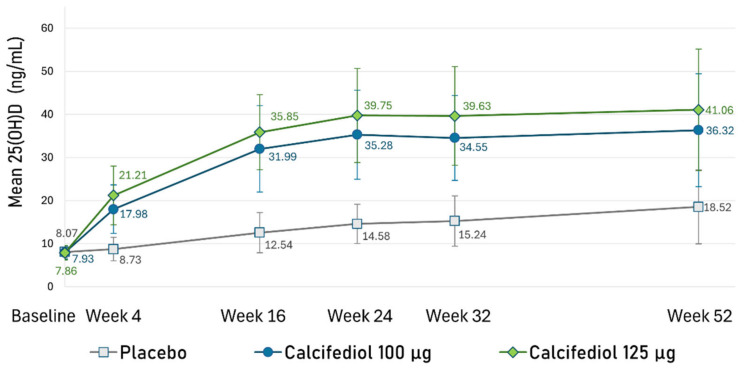
Mean plasma 25(OH)D concentrations (ng/mL) at each designated time point are illustrated for the placebo group (grey line), 100 µg calcifediol group (dark blue line), and 125 µg calcifediol group (green line). Error bars represent standard deviations. A *p*-value < 0.02 was observed at every visit for comparisons between active treatment groups, while a *p*-value < 0.0001 was observed when comparing placebo to active treatments at each visit.

From week 24 onwards, serum 25(OH)D levels remained nearly stable in both calcifediol groups through to week 52 (Figure 6). The mean (SD) steady state 25(OH)D level was 35.37 (11.13) ng/mL in the 100 µg calcifediol group and 40.13 (12.18) ng/mL in the 125 µg calcifediol group.

### 3.6. Safety

The occurrence of Treatment Emergent Adverse Events (TEAEs) was similar in the placebo and the calcifediol groups (Table 2). A total of 95 (34.9%) subjects experienced 190 TEAEs. Nine subjects (3.3%), which were mainly observed in the placebo group (seven subjects), experienced a TEAE assessed as related to treatment. Most TEAEs were of mild or moderate severity, although severe TEAEs were reported in eight (2.9%) subjects. Overall, nine (3.3%) subjects prematurely discontinued from the study or withdrew from treatment due to a TEAE. Except for upper abdominal pain, which was considered possibly related to calcifediol 125 µg, all other TEAEs that lead to discontinuation were assessed as unrelated to treatment. Two deaths occurred during the study, caused by COVID-19 pneumonia and sudden death, and a cardiogenic shock was reported 44 days after the last intake of trial medication, but all were considered not related to treatment. The frequency of serious TEAEs, mainly infections, was three events in the placebo group, three in the calcifediol 100 µg group and five in the calcifediol 125 µg group. None of them were considered related to treatment. No SUSARs were reported during the study (Table 2).

The observed average changes in bone mineral parameters at weeks 16 and 52 are presented in Table 3. It can be observed that the reductions in PTH values from baseline were greater in the calcifediol groups compared to the placebo group. On the other hand, the mean change from baseline in total serum calcium was similar across all treatment groups (Table 3). Elevated total serum calcium, considered as values ≥10.5 mg/dL, was reported in eight subjects: three in the placebo arm (5.5%), two in the calcifediol 100 µg arm (1.9%), and three in the calcifediol 125 µg arm (2.7%). Maximum value reached was 11.3 mg/dL. There was no correlation between levels of 25(OH)D and calcium (r = 0.235, *p* = 0.439).

In the calcifediol 100 µg group, there were 5 subjects (4.8%) who reached 25(OH)D levels above 60 ng/mL, compared to 12 subjects (10.6%) in the calcifediol 125 µg group. Only one subject of the calcifediol 125 µg arm, at week 52, reached 25(OH)D levels above 80 ng/mL (84.72 ng/mL).

## 4. Discussion

Vitamin D is essential for the effective absorption of calcium, magnesium, and phosphate within the human body [22]. But it also performs numerous additional biological roles, including the maintenance of bone integrity and the support of muscular, neural, and immune system functions [23]. Although vitamin D deficiency is often asymptomatic, patients with prolonged and severe vitamin D deficiency may develop symptoms related to secondary hyperparathyroidism. This includes bone pain, arthralgia, myalgia, fatigue, muscle twitching (fasciculations), and weakness [24]. The widespread prevalence of vitamin D deficiency [25] and the associated health risks highlight the importance of supplementation, especially for individuals with severe deficiency.

The phase II/III, randomised, double-blind, placebo-controlled clinical trial showed in this manuscript, was conducted to evaluate the efficacy and safety of new different strengths of weekly dose formulations of calcifediol in patients with severe vitamin D deficiency, i.e., 25(OH)D ≤10 ng/mL]. These patients were randomised to receive weekly placebos or calcifediol at doses of 100 µg or 125 µg and assessed for safety and efficacy outcomes. The results demonstrate that both doses effectively raised serum 25(OH)D to adequate levels, with good safety and tolerability. Notably, the equivalent efficacy observed between the weekly doses of 100 µg and 125 µg, combined with the more favourable long-term safety profile associated with the lower dose, suggests that weekly 100 µg of calcifediol may be the best treatment option. This high efficacy and safety observed for weekly calcifediol 100 µg in the treatment of severe vitamin D deficiency was also previously described for the treatment of a less severe form of the pathology in the assessment of patients with 25(OH)D plasma levels between 10 and 20 ng/mL [20].

Recent Clinical Practice Guidelines co-sponsored by the American Association of Clinical Endocrinology (AACE) and the European Society of Endocrinology (ESE), among others, highlighted a shortage of clinical trials on vitamin D supplementation in severely deficient populations [13]. The present study addresses this gap by focusing on patients with severe vitamin D deficiency.

The results of this study showed that weekly supplementation raised mean 25(OH)D levels to the optimal 30 ng/mL by week 16 with levels stabilizing at week 24 and maintained through week 52. This rapid and sustained response to supplementation, characterised by an initial increase followed by a plateau, aligns with findings from several previous studies evaluating the efficacy and pharmacokinetics of calcifediol in monthly, biweekly, or daily dosing regimens (reviewed in [26,27]. All this clinical trials obtained a notable efficacy of calcifediol in achieving 25(OH)D levels above optimal 30 ng/mL, with higher levels being reached at higher doses of treatment, which is also in line with our results [28,29]. Previous trials have also demonstrated calcifediol’s efficacy in restoring 25(OH)D levels in vitamin D-deficient patients, as well as the impact of this restoration on pre-existing pathologies. They observed an improvement in physical performance, muscle strength, asthma control, and quality of life, as well as its potential benefits in managing conditions like COVID-19 complications and decompensated cirrhosis [30,31,32,33,34,35,36,37], demonstrating the advantages of calcifediol supplementation and the potential of a formulation indicated for weekly treatment in the clinical settings.

Wimalawansa in 2022 concluded that rapid repletion of vitamin D can significantly enhance immune function and reduce the risk of complications following infections [17]. This suggests the potential clinical benefits of the rapidity in the increase in 25(OH)D levels that was also observed after weekly calcifediol administration, particularly for patients with severe deficiency.

Weekly administered doses of 100 µg and 125 µg of calcifediol have shown a good safety profile consistent with previous studies [38,39,40]. The most frequent consequence of vitamin D toxicity is hypercalcemia [38]. Lee et al. [41] reviewed data from 127,932 samples collected from 73,779 patients over 16 years at a university hospital. They found that 1.1% (780 subjects) had 25(OH)D levels above the safety threshold of 80 ng/mL, and only seven patients had elevated total calcium [t(Ca)], of whom four exhibited clinical symptoms. A correlation between t(Ca) and 25(OH)D levels was observed starting from a specific 25(OH)D threshold, as all patients with elevated t(Ca) had 25(OH)D levels above 100 ng/mL. When hypercalcemia was symptomatic, 25(OH)D levels exceeded 150 ng/mL. In the present study, no subject reached the 100 ng/mL threshold for 25(OH)D, and patients with t(Ca) levels ≥10.5 mg/dl did not present elevated 25(OH)D levels. Thus, no association between elevated t(Ca) and 25(OH)D levels was observed. Furthermore, the highest percentage of patients with elevated serum t(Ca) levels was observed in the placebo group.

The main limitation of this study was the lack of an active comparator, such as cholecalciferol or other calcifediol formulations. However, the study was placebo-controlled to account for seasonal fluctuations in endogenous 25(OH)D levels. The intended sample size was not achieved due to the influence of SARS-CoV2 infection. Additionally, this study did not measure hypercalciuria although it is related with higher levels of total serum calcium than observed in this trial, i.e., 12 mg/dL [42]. Finally, the corrected calcium for albumin levels was not estimated. A key strength of this study is its sample size of 466, making it one of the largest studies on calcifediol in patients with severe vitamin D deficiency with a remarkable long follow-up. It is also important to note that over 80% of participants completed the study, and treatment compliance was very high, exceeding 90% at week 52 in all treatment groups. The long duration (12 months) of the study, which allows each patient to begin and end the study in the same season, and the centralization of laboratory analyses were also strengths of the present study.

## 5. Conclusions

Calcifediol has consistently been shown to be an effective, predictable, and safe option for prevention and treatment of vitamin D deficiency [13,26]. Overall, this trial showed that the formulations of 100 µg and 125 µg calcifediol once a week are highly effective in achieving adequate serum 25(OH)D concentrations in a rapid and safe manner. Given the comparable long-term efficacy of both weekly doses of calcifediol, the weekly 100 µg calcifediol dose may represent the most appropriate treatment option for patients with severe vitamin D deficiency. Determining the appropriate dose for weekly treatment provides a new therapeutic formulation option for vitamin D deficient patients in clinical practice. Additionally, the weekly regimen has the potential to increase treatment adherence and compliance.

## Figures and Tables

**Table 1:** Demographics and main previous comorbidities. BMI, body mass index; n, number of subjects; SD, standard deviation.

Parameter	Placebo	Calcifediol 100 µg	Calcifediol 125 µg	Total
	(n = 55)	(n = 104)	(n = 110)	(n = 269)
Age, mean (SD) years	53.8 (12.9)	55.4 (16.0)	55.7 (16.1)	55.2 (15.4)
Sex: female, n (%)	43 (78.2)	79 (76.0)	76 (69.1)	198 (73.6)
BMI, mean (SD) kg/m^2^	30.5 (8.3)	29.3 (7.2)	29.5 (6.6)	29.7 (7.2)
BMI, n (%):				
<18.5 kg/m^2^	0	2 (1.9)	3 (2.7)	5 (1.9)
≥18.5 <25 kg/m^2^	19 (34.5)	29 (27.9)	24 (21.8)	72 (26.8)
≥25 <30 kg/m^2^	12 (21.8)	30 (28.8)	37 (33.6)	79 (29.4)
≥30 kg/m^2^	24 (43.6)	42 (40.4)	46 (41.8)	112 (41.6)
Main comorbidities:				
Hypertension, n (%)	28 (50.9)	52 (50.0)	53 (48.2)	133 (49.4)
Menopause, n (%)	21 (38.2)	49 (47.1)	46 (41.8)	116 (43.1)

**Table 2:** Overview of TEAEs. The table includes the percentage (%) and number (n) of patients in the safety population of each group (N) who experienced “E” events.

	Placebo		Calcifediol		Calcifediol		Total	
	(N = 55)		100 µg (N = 104)		125 µg (N = 113)		(N = 272)	
	n (%)	E	n (%)	E	n (%)	E	n (%)	E
TEAE	21 (38.2)	47	33 (31.7)	62	41 (36.3)	81	95 (34.9)	190
Non-serious TEAE	21 (38.2)	44	32 (30.8)	59	40 (35.4)	76	93 (34.2)	179
Serious TEAE	3 (5.5)	3	3 (2.9)	3	4 (3.5)	5	10 (3.7)	11
Related TEAE	7 (12.7)	7	0	0	2 (1.8)	2	9 (3.3)	9
Related serious TEAE	0	0	0	0	0	0	0	0
Severe TEAE	3 (5.5)	3	3 (2.9)	3	2 (1.8)	3	8 (2.9)	9
TEAE leading to discontinuation	1 (1.8)	1	2 (1.9)	2	6 (5.3)	6	9 (3.3)	9

**Table 3:** Overview of changes at weeks 16 and 52 in bone and mineral metabolism parameters. The table indicates total Safety Set patients (N), those with available data (n), and the mean parameter value with the standard deviation (SD).

Parameter Visit	Placebo (N = 55)		Calcifediol 100 µg (N = 104)		Calcifediol 125 µg (N = 113)	
	n	Mean (SD)	n	Mean (SD)	n	Mean (SD)
Alkaline phosphatase (U/L)						
Week 16	54	2.3 (13.03)	102	−3.8 (17.22)	105	−9.1 (26.29)
Week 52	41	−5.7 (27.67)	91	−4.4 (13.93)	93	−7.8 (21.16)
Total serum Ca (mg/dL)						
Week 16	53	0.13 (0.336)	102	0.09 (0.351)	105	0.12 (0.407)
Week 52	40	0.15 (0.399)	91	0.11 (0.384)	93	0.16 (0.352)
Phosphorous (nmol/L)						
Week 16	54	0.034 (0.178)	103	0.025 (0.180)	105	0.022 (0.239)
Week 52	41	0.054 (0.189)	92	0.049 (0.191)	93	0.015 (0.226)
Parathyroid hormone (pg/mL)						
Week 16	50	−8.3 (20.32)	100	−14.3 (20.19)	101	−16.1 (22.73)
Week 52	37	−8.6 (19.29)	90	−15.6 (19.06)	89	−15.8 (21.69)

## Data Availability

The original contributions presented in this study are included in the article and Supplementary Materials. Further inquiries can be directed to the corresponding author.

## Supplementary Materials

The following supporting information can be downloaded at www.mdpi.com/article/10.3390/nu17040672/s1: Table S1: Schedule of events and assessments by visit; Table S2: Inclusion and exclusion criteria; Table S3: Materials and Methods citations; Table S4: Number and percentage of subjects by season at the start of treatment; Figure S1: Proportion of responders by BMI subgroup.
