# Supplementary material for: A Randomized Phase II/III Trial Evaluating the Efficacy and Safety of 100 and 125 µg of Calcifediol Weekly Treatment of Severe Vitamin D Deficiency
Source: Nutrients. 2025 Feb 13;17(4):672. doi: 10.3390/nu17040672 (PMC11857939; doi:10.3390/nu17040672)

**- SUPPLEMENTARY INFORMATION –**

for

# A randomized phase II/III trial evaluating the efficacy and safety of 100 and 125 µg of calcifediol weekly treatment of severe vitamin d deficiency

by Pérez-Castrillón et al.

**Table S1**. Schedule of events and assessments by visit.

| | SCREENING | RANDOMISATION | TREATMENT PERIOD[1] | | | | | FOLLOW-UP |
|---|---|---|---|---|---|---|---|---|
| | | | MAIN PHASE | | EXTENSION PHASE | | | |
| | | | 4 weeks | 16 weeks | 24 weeks | 32 weeks | 52 weeks | 55-56 weeks |
| | Within 10 days before randomisation | Within 6 to 2 days before first IP intake[2] | Days 24-27 | Days 108-111 | Days 164-167 | Days 220-223 | Days 360-363/ EDV | Days 388-398 |
| | V1 | V2 | V3 | V4 | V5 | V6 | V7 | V8 |
| In-house visit ⊗ / telephone contact ☎ | ⊗ | ⊗ | ⊗ | ⊗ | ⊗ | ⊗ | ⊗ | ☎ |
| Informed consent | X | | | | | | | |
| Medical history | X | | | | | | | |
| Prior medication | X | | | | | | | |
| Concomitant medication | X | X | X | X | X | X | X | |
| Calcium consumption questionnaire | | X | X | X | X | X | X | |
| Record demographic information and height | X | | | | | | | |
| Review inclusion/exclusion criteria | X | X | | | | | | |
| Randomisation | | X | | | | | | |
| Dispense IP | | X | | X | | X | | |
| Dispense rescue medication | | | | X[3] | X[3] | X[3] | | |
| Collect IP | | | | X | | X[5] | X[5] | |
| Drug accountability (IP and rescue medication) | | | | X | | X | X | |
| Blood sample (25-OH-D) | X | | X | X[4] | X | X | X | |
| Review of adverse events (n-TEAEs or TEAEs) | X | X | X | X | X | X | X | X |
| Blood sample (haematology, biochemistry, bone and mineral metabolism) with prior 8-hour fasting | X | | X | X | X | X | X | |
| Blood pregnancy test (serum-hCG) | X | | | | | | X | |
| Urine pregnancy test | | X | X | X | X | X | | |
| Vital signs | X | | | X | | | X | |
| Physical examination | X | | | X | | | X | |
| Body weight, abdominal circumference | | X | | X | X | | X | |

1. In all cases, visits that require blood sampling should be performed at least 48 hours after last IP intake.
2. IP intake will take place on the first Sunday after randomisation visit (V2), and that will be considered as Day 1. Days in the table are related to IP intake. Last IP intake (and last rescue medication intake, if applicable) is expected on Day 358.
3. If the subject has 25-OH-D levels $\leq$ 10 ng/mL, rescue medication (Benferol® 800 I.U./daily) will be provided at an unscheduled visit 1-2 weeks later.
4. Primary efficacy analysis.
5. If rescue medication was provided, it will also be collected.

**Table S2.** Inclusion and exclusion criteria.

| | |
|---|---|
| **Inclusion criteria** | 1. Male or female subjects ≥ 18 years of age.<br><br>2. Evidence of serum 25(OH)D levels < 20 ng/mL or ≤ 10 ng/mL, for each cohort, respectively.<br><br>3. Written informed consent given freely after the nature of the clinical study and disclosure of data has been explained to the subject.<br><br>4. For females of childbearing potential only: willing to perform pregnancy tests and had to agree to use highly effective methods of birth control throughout the study, from the time of informed consent signature until 30 days after last IP intake. Highly effective methods of birth control included combined hormonal contraception associated with inhibition of ovulation (oral, intravaginal or transdermal), progestogen-only hormonal contraception associated with inhibition of ovulation (oral, injectable or implantable), intrauterine device, intrauterine hormone-releasing system, bilateral tubal occlusion, vasectomized partner (provided that partner is the sole sexual partner of the clinical study participant and has documentation of azoospermia) or sexual abstinence (if defined as refraining from heterosexual intercourse during the entire period of risk associated with the study treatment). The investigator was responsible for determining whether the subject had adequate birth control for study participation.<br><br>4. (For France only): Confirmation of affiliation to a social security system by signing the informed consent form. |
| **Exclusion criteria** | 1. Subjects receiving any treatment with calcifediol, vitamin D analogues, vitamin complexes or vitamin D supplements within the last week before screening or planned during the clinical study (except clinical study rescue medication).<br><br>2. Subjects taking drugs that could modify vitamin D levels, i.e., phenobarbital, phenytoin, primidone, digoxin, rifampin, thiazide diuretics (hydrochlorothiazide), some antibiotics (penicillin, neomycin and chloramphenicol), antiretrovirals (tenofovir, adefovir), long-term corticosteroids (defined as a dose of prednisolone ≥ 5 mg per day (or equivalent) for more |

| | |
|---|---|
| | than 3 months), verapamil, paraffin, mineral oil laxatives, magnesium salts, actinomycin, and antifungal imidazoles, within the last week before screening or was planned during the clinical study. Subjects taking orlistat, cholestyramine or colestipol who did not respect an interval of at least 2 hours before IP intake.<br><br>3. Subjects taking calcium supplements within the last week before screening or planned during the clinical study.<br><br>3a. (For Czech Republic): Subjects with confirmed osteoporosis.<br><br>4. Uncorrected hypercalcaemia (calcium > 10.5 mg/dL), known hypercalciuria or nephrolithiasis.<br><br>4a. (For Czech Republic): Subjects aged 40 years or older with associated risk factors for osteoporosis determined by the Fracture Risk Assessment Tool (FRAX) if the ten-year risk of major osteoporotic fracture was ≥ 20% OR if the ten-year risk of hip fracture was ≥ 3%. In subjects younger than 40 years old, risk factors for osteoporosis were to be assessed according to local guidelines. Subjects could only be included if their risk was negligible, and their overall evaluation was considered adequate in the investigator's opinion. Exception: In both cases, subjects could be included if a dual-energy X-ray absorptiometry (DXA) scan performed within 12 months before Visit 1 with normal age-appropriate bone mineral density results in the lumbar spine and total proximal femur was available.<br><br>5. Severe renal impairment, defined as an estimated glomerular filtration rate (eGFR) by CKD-EPI < 30 mL/min/1.73 m2.<br><br>6. Subjects diagnosed with liver or biliary failure, congestive heart failure, malabsorption, primary hyperparathyroidism, hypothyroidism, prolonged immobilization, sarcoidosis, tuberculosis or other granulomatous diseases or hyperthyroidism.<br><br>7. Any present or previous malignancy within the last 5 years prior to the screening visit. |

| | |
|---|---|
| | 8. Known contraindications or sensitivities to the use of the IP or any of its components.<br><br>9. Pregnant woman, breastfeeding woman or woman planning a pregnancy.<br><br>10. Subject had received an IP (including investigational vaccines) or used an invasive investigational medical device within 30 days (or five half-lives of that IP, whichever is longer) before the start of the screening or was currently enrolled in an investigational interventional study.<br><br>11. Any condition that, in the opinion of the investigator, might jeopardize the clinical study conduct according to the protocol (for example, evidence of diseases, medications or laboratory abnormalities that could alter the conduct of the study).<br><br>12. Employees of the investigator or clinical study site, with direct involvement in the proposed study or other studies under the direction of that investigator or clinical study site, as well as family members of the employees or the principal investigator.<br><br>13. Person committed to an institution by virtue of an order issued either by judicial or other authorities.<br><br>14. (For France): Adult subjects with incapacities under legal representation according to national laws. |

**Table S3.** Materials and Methods citations.

| | |
|---|---|
| **LKF** | Laboratorium für Klinische Forschung GmbH, Schwentinental, Germany |
| **Immunoassay system** | Elecsys Vitamin D Total II assay, Roche Diagnostics GmbH, Mannheim, Germany |
| **SAS Software** | Statistical Analysis System (SAS® version 9.4 for Windows, Institute Inc., Cary, NC, USA) |
| **nQuery Software** | nQuery Advanced software 8.2 (Statistical Solutions Ltd., Cork, Ireland) |

| | |
|---|---|
| **Rescue medication** | Benferol® 800 IU soft capsules, Consilient Health Limited |

**Table S4.** Number and percentage of subjects by season at the start of treatment.

| Season at start of treatment | Placebo (N=55) | | Calcifediol 100µg (N=104) | | Calcifediol 125µg (N=110) | | Total (N=269) | |
|---|---|---|---|---|---|---|---|---|
| | n | % | n | % | n | % | n | % |
| Winter | 15 | 27.3 | 31 | 29.8 | 32 | 29.1 | 78 | 29.0 |
| Spring | 31 | 56.4 | 57 | 54.8 | 52 | 47.3 | 140 | 52.0 |
| Summer | 3 | 5.5 | 10 | 9.6 | 17 | 15.5 | 30 | 11.2 |
| Fall | 6 | 10.9 | 6 | 5.8 | 9 | 8.2 | 21 | 7.8 |

**Figure S1.** Proportion of responders by BMI subgroup for level ≥20 ng/mL (a) or ≥30 ng/mL (b) at week 16. Estimates for differences in proportions (p-values) are based on a two-sided test of proportions using normal approximation with significance level 0.05. n, number of subjects; %, percentage based on n.

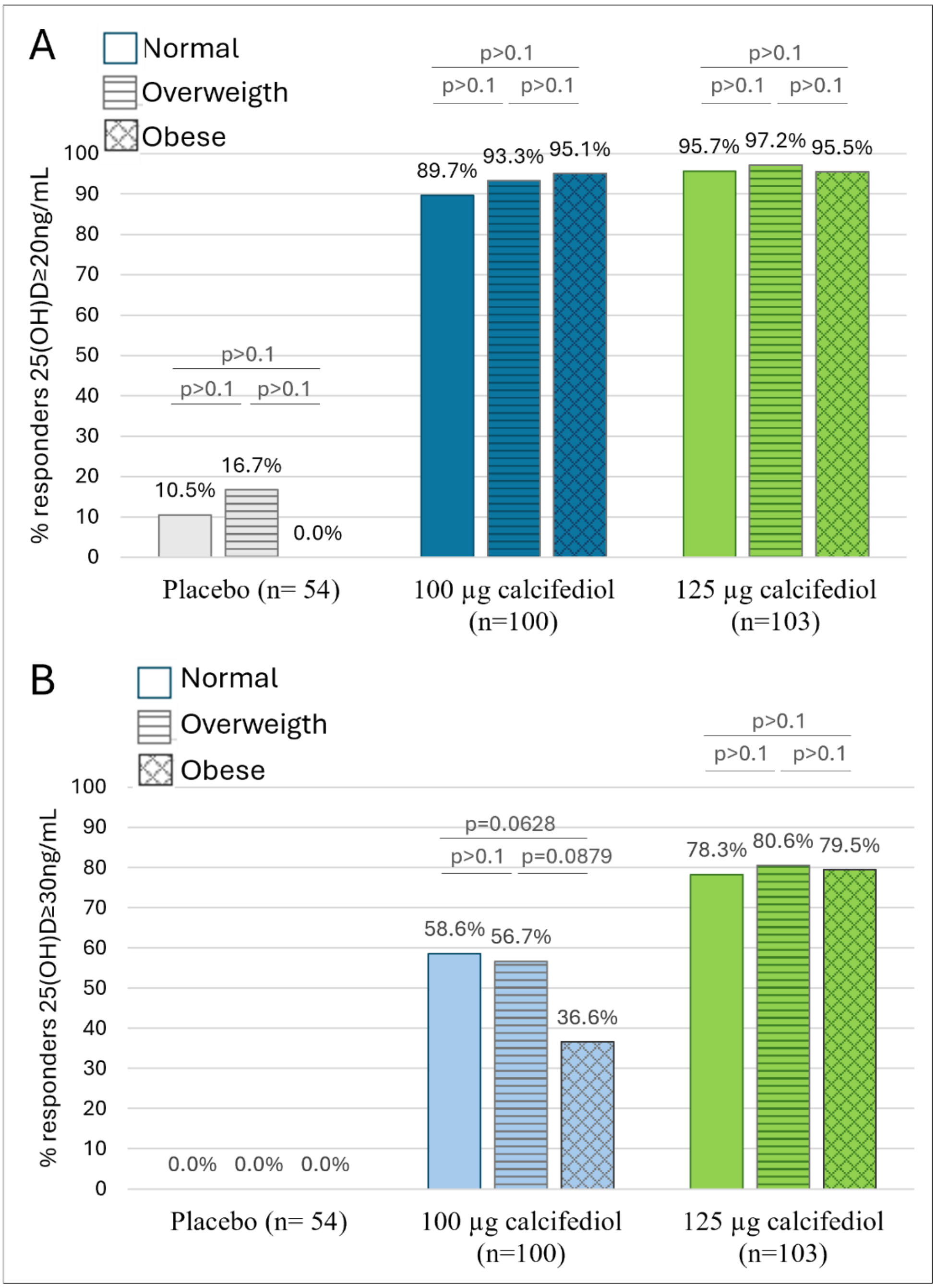

Supplement: Supplementary file 1 [file nutrients-17-00672-s001.zip › nutrients-3396083-supplementary.pdf]
